# MiR-signing gastrointestinal (con-)tract cancer

**DOI:** 10.18632/oncotarget.15701

**Published:** 2017-02-25

**Authors:** Vassilis G. Gorgoulis, Ioannis S. Pateras, Athanassios Kotsinas

**Affiliations:** Department of Histology and Embryology, School of Medicine, National Kapodistrian University of Athens and Biomedical Research Foundation of the Academy of Athens, Athens, Greece and Faculty of Biology, Medicine and Health, University of Manchester, Manchester Academic Health Science Centre, Manchester, UK

**Keywords:** miR204, FOXM1, gastrointestinal tumors, Epithelial-Mesenchymal-Transition, DNA-damage-response

MicroRNAs (miRs) are evolutionary conserved, small non-coding RNAs with a crucial role in regulating genes expression [1]. As they target simultaneously several genes, one miR can eventually control a variety of cellular functions. Therefore, aberrations in the expression of miRs have detrimental repercussions for disease development and progression, including cancer.

In the current issue, the Blandino group elegantly demonstrated that mir-204 is significantly downregulated in tumors of the gastrointestinal tract [2]. Mir-204 is deregulated in many types of cancer, exhibiting a tumor-suppressor role in the majority of them. Although evidence has implicated mir-204 in the development of gastric and esophageal cancer, a systematic *in vivo* analysis to define its targets and address the functional consequences from escaping mir-204 suppression was missing in tumors of the gastrointestinal tract [1].

**Figure 1:**
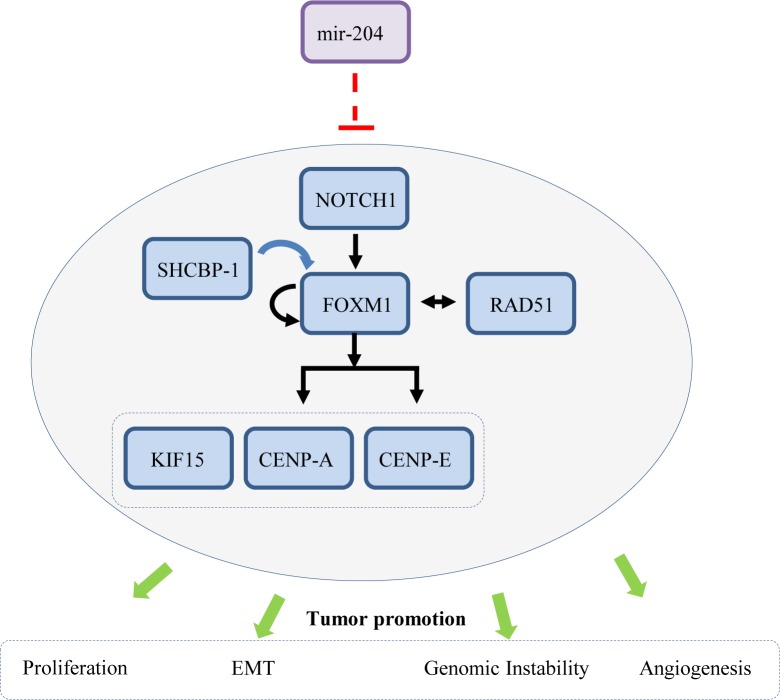
Mir-204 regulates a multi-layered cellular signaling network [CENP-A (Centromere Protein A), SHCBP-1 (SHC Binding And Spindle Associated 1), FOXM1 (Forkhead Box M1), KIF15 (Kinesin Family Member 15), CENP-E (Centromere Protein E), RAD51 (DNA repair protein RAD51 homolog 1) and NOTCH1 (Neurogenic locus notch homolog protein 1)] black arrows: direct effect; blue arrow: indirect effect; green arrows: cellular effect

Focusing in parallel into gastric cancer and cholangiocarcinoma, a rare tumor type of the digestive system, Canu and collaborators provide evidence that mir-204 downregulation is associated with a specifically altered 7-gene expression signature [2]. *In silico* and functional analyses demonstrated that all 7 genes (Figure 1) (CENP-A, SHCBP-1, FOXM1, KIF15, CENP-E, RAD51 and NOTCH1) in this signature were direct targets of mir-204, each influencing in an additive manner cell cycle progression and clonogenicity in corresponding cellular systems.

At a first glance all seven genes seem to be almost unrelated, yet a closer examination reveals a potential synergy between them, providing a functional frame for these findings. The most central, orchestrating, gene seems to be FOXM1. It is a transcription factor that stimulates S and M phase entry and is overexpressed in many cancer signatures. Notably, among the many factors that it upregulates, are also CENP-A, CENP-E, RAD51 and itself, thus forming a positive feedback loop. In turn NOTCH1, a key differentiation factor, as well as RAD51 upregulate FOXM providing an additional layer to sustain the mir-204 signature [3].

Both FOXM1 and NOTCH1 exert many tumor promoting capabilities, such as augmenting proliferation, anchorage-independent growth, angiogenesis and epithelial to mesenchymal transition (EMT) [3]. While Canu and collaborators have documented increased proliferation, it would be interesting to explore if this signature promotes also other tumorigenic features in gastronintestinal cancers. Notably, FOXM1 induces EMT in esophageal cancer upon loss of mir-204 [1]. Together with NOTCH1 they can promote EMT by upregulating classical EMT inducers that suppress E-cadherin. Intriguingly, FOXM1 also promotes expression of the replication licensing factor CDC6, which when overexpressed can repress E-cadherin and trigger genomic instability [3, 4]. In a RAS activated background CDC6 mediated E-cadherin suppression results in EMT [4]. It would be interesting to define whether such a scenario takes place in gastric tumors and cholangiocarcinomas, too. Toward this direction is the fact that FOXM1 activates the RAS/MAPK pathway, which in turn induces Cdc6 expression [3, 4]. The picture is further enriched by SHCBP-1, a member of the mir-204 signature, which is an adaptor of the SHC protein participating in the receptor tyrosine kinases signaling of RAS [3].

The above landscape becomes more complex given that NOTCH1 can partially impair the DNA damage response (DDR) pathway by suppressing ATM [5]. If such an event takes places in gastrointestinal tumors then ATM downregulation should activate the ARF anti-tumor barrier [6], unless ARF is suppressed by CDC6, which is frequently overexpressed in colorectal cancer from its early stages [4]. It would be interesting to explore this hypothesis.

Finally, CENP-A, CENP-E and KIF15, are key factors of mitosis. CENP-E and KIF15 are kinesin-like motor enzymes involved in mitotic spindle assembly, while CENP-A is a histone H3-like nucleosomal protein that is present in centromeric nucleosomes and is involved in recruitment and assembly of the kinetochore. The high levels of these factors in the mir-204 signature, possibly reflect an increased segregation demand due to genomic instability. Within this frame, RAD51 is increased and FOXM1 deregulates the replication licensing machinery, features that are linked with the emergence of genomic instability [3, 7].

Overall the results from Canu and collaborators pinpoint the complexity that may emerge from aberrations of a single miR. Mir-204 seems to control a multi-layered cellular signaling network (Figure 1), setting new questions that need to be addressed. One regards, how early does loss of mir-204 takes place during the development of these tumor types? It is reported that the majority of miRs rely within fragile sites, known to be affected very early during cancer development [8]. As mir-204 is located at 9q21.12 chromosomal locus that does not seem to comprise a fragile site [1], what mechanism(s) underlie its deregulation? If loss of mir-204 expression is eventually an early driving event in gastrointestinal cancer, it may provide a promising target for future therapeutic interventions.
